# Editorial: Considering Biological Sex in Neurological Research

**DOI:** 10.3389/fneur.2021.698492

**Published:** 2021-07-02

**Authors:** Anat Biegon, Erin Sundermann

**Affiliations:** ^1^Radiology and Neurology, Stony Brook University, Stony Brook, NY, United States; ^2^University of California, San Diego Health, San Diego, CA, United States

**Keywords:** male–female distinctiveness, sex, cognitive function, personalized medicine, gender

Biological sex, gender, and gonadal hormone status have a substantial influence on the incidence, prevalence, presentation, treatment response, and outcome of diverse neurological/neuropsychological disorders and pathologies. Despite increasing recognition of the importance of these variables, demographic data collected from patients and participants in clinical trials and basic research do not routinely include information on gonadal hormone status (e.g., puberty, stage of menstrual cycle, menopause, or andropause) and results are often not stratified by sex or gonadal hormone status. In this special Issue, investigators and practitioners engaged in research and treatment of diverse neuropsychological issues in human subjects and animal models share their experimental results and observations regarding the impact of biological sex and gonadal hormones on cognitive performance in the context of diabetes, HIV, menopause, obesity and Parkinson's disease. Other contributions examine the role of biological sex and hormonal status in Huntington's disease, early-stage Alzheimer's disease, migraine, epilepsy, stroke, and traumatic brain injury.

Neurocognitive function in the context of prediabetes is addressed by Sundermann et al. who examined sex differences in how pre-diabetic status relates to trajectories of Alzheimer's-related clinical (verbal memory, executive function and language performance and dementia incidence) and biological (brain metabolism, hippocampal volume, cerebrospinal fluid p-tau_181_/Aβ_1−42_ ratio) markers among cognitively normal and mildly cognitively impaired (MCI) older adults from the Alzheimer's Disease Neuroimaging Initiative (*N* = 911, 46% women). Whereas, pre-diabetes shows adverse effects on brain metabolism across sexes, only women with MCI showed associations between pre-diabetes and poorer executive function and language performance across time suggesting that women may be more susceptible to the negative effects of pre-diabetes on cognition. Using machine learning methods, *Rubin et al.* investigated how profiles of neurocognitive dysfunction among persons with HIV and their associated risk/protective factors differ by sex. Whereas, men with HIV showed an unimpaired profile and even a cognitively advantageous profile, women with HIV only showed impairment profiles that included global and domain-specific impairment. Meanwhile, the most discriminative risk/protective factors (e.g., reading level, age, and HIV disease characteristics) were similar across sexes. Results suggest that sex is a major contributor to the heterogeneity in cognitive impairment profiles in HIV.

In a study focusing on menopause, Maki and Thurston review the emerging literature on importance of examining menopausal symptoms, in addition to estradiol effects, as a mechanism contributing to cognitive and brain aging in women. The authors highlight the utility of objective measures of menopause symptoms, particularly vasomotor symptoms, in assessing associations with memory performance, brain structure and brain function.

Two papers describe the cognitive effects of gonadal hormone withdrawal and replacement in animal models: Zimmerman et al. examined cognitive performance and hippocampal volume in menopausal macaques fed a high-fat, high sugar diet and exposed to either immediate or delayed treatment with estradiol. The authors find beneficial effects of the delayed estradiol treatment on spatial memory and hippocampal volume relative to placebo. Conner et al. examined the effects of sex and gonadal hormones on episodic-like memory in a rat model of early (premotor) Parkinson's disease and report that male gonadectomy ameliorated memory deficits induced by the model in a domain-specific manner. The effect of orchiectomy was completely reversed by testosterone but estradiol had no effect, suggesting direct effects testosterone acting via androgen receptors.

Effects of sex and gonadal hormones on disease presentation are addressed in an opinion piece by Zielonka and Stawińska-Witoszyńska, where the importance of research in the underexplored topic of sex/gender differences in non-sex linked disorders is brought to light. As an example, they discuss the emerging evidence of sex/gender differences on the progression rate and clinical features pattern of Huntington's disease.

In a systematic review, Al-Hassany et al. surveyed existing findings regarding sex and gender differences in migraine and synthesized findings across domains of biological/pre-clinical, clinical, and population-level research. Important knowledge gaps are identified and priorities are set for further research in sex and gender differences in migraine and in therapeutic options.

The impact of sex and hormonal status on clinical management is addressed by Spiegel and Merius in the context of epilepsy, reviewing accumulating knowledge on the teratogenic effects of anti-seizure medications and the effects of gonadal hormones on the pharmacokinetic profile of these drugs and how it is translated into specific guidelines for the treatment of females before and after puberty and during pregnancy.

Two papers focus on sex differences in outcome following ischemic or traumatic injury to the brain: Scrutinio et al. address sex differences in long-term mortality and functional outcome after severe stroke. In this British cohort of 1,316 subjects (mean age 72, 44% women), they find lower stroke—related mortality in women relative to men but no advantage in functional recovery. A mortality advantage in women over 50 (presumably post-menopausal) relative to men in the same age group is also described in a minireview of sex differences in traumatic brain injury (Biegon). Studies reviewed also suggest that young (presumably pre-menopausal) women do not share this advantage and actually tend to have a worse outcome following mild concussive injuries to the brain. This minireview also highlights the very slow adaptation of sex as a biological variable in the design and analysis of clinical trials: Between 1996 and 2020, the percentage of women in key clinical trials in TBI was consistently below 30% and only one study, published in 2014, stratified outcome by sex.

This collection of studies and reviews exemplifies the continuity of sex/gender differences across different types of neurological disease and the importance of accounting for these differences in research. These findings also illustrate how endocrine events throughout the lifespan (e.g., menstrual cycle, menopause, pregnancy) may influence these sex differences in disease outcomes. As the field shifts toward personalized medicine, findings such as these create a platform for future studies to advance the development of risk assessments and diagnostic and therapeutic strategies that are optimized and personalized by sex and hormonal status. Moreover, discovery of differences in disease outcomes by sex and gonadal hormone status can serve as a window into causal pathways of disease and therapeutic targets, thus, enhancing our overall understanding of disease and their treatment in all.

## Author Contributions

All authors listed have made a substantial, direct and intellectual contribution to the work, and approved it for publication.

## Conflict of Interest

The authors declare that the research was conducted in the absence of any commercial or financial relationships that could be construed as a potential conflict of interest. The Handling Editor HC declared a shared affiliation with one of the authors ES at the time of the review.

